# Development of a transformation model to analyze horizontal saccadic velocity using electrooculography: a pilot feasibility study

**DOI:** 10.3389/fnins.2026.1862069

**Published:** 2026-07-07

**Authors:** Da Young Kim, Tae-Joon Kim, Yunsoo Kim, Jisu Yoo, JaeWook Jeong, Sun-Uk Lee, Jun Young Choi

**Affiliations:** 1 Department of Neurology, Ajou University Hospital, Suwon, Republic of Korea; 2 Department of Convergence Healthcare Medicine, Ajou University, Suwon, Republic of Korea; 3 Department of Neurology, Ajou University School of Medicine, Suwon, Republic of Korea; 4 Neurotology and Neuro-Ophthalmology Laboratory, Korea University Medical Center, Seoul, Republic of Korea; 5 Department of Neurology, Korea University Medical Center, Seoul, Republic of Korea; 6 Department of Brain Science, Ajou University School of Medicine, Suwon, Republic of Korea

**Keywords:** biomarkers, electrooculography, eye movements, saccades, videooculograph(y) (VOG)

## Abstract

**Introduction:**

Saccadic eye movements are established biomarkers in neuroscience and clinical neurology, with video-oculography (VOG) serving as the gold standard for measurement. However, the high cost, bulky equipment, and poor portability of VOG systems restrict their clinical utility. Electrooculography (EOG) provides a practical alternative, but quantitative conversion of EOG-derived measurements into VOG-equivalent values remains insufficiently established. This study aimed to develop and validate a mathematically derived transformation model for estimating VOG-equivalent horizontal saccadic velocities from EOG recordings.

**Methods:**

Four healthy adults underwent simultaneous EOG and VOG recordings while performing controlled horizontal gaze shifts. Based on a current-source model of the corneal potential, an analytical relationship between EOG voltage velocity and angular eye velocity was derived. Multiple high-pass filter settings were systematically evaluated to identify optimal signal-processing conditions. Transformation equations were derived from the pooled horizontal-saccade dataset and further evaluated using leave-one-subject-out (LOSO) analysis.

**Results:**

The theoretical model predicted a linear relationship between EOG- and VOG-derived saccadic velocities. Among the tested filter settings, a 0.3 Hz high-pass combined with a 35 Hz low-pass filter yielded the best overall agreement. Under this condition, the final transformation model produced a common slope coefficient of 0.146 °/μV for both movement directions, with an additional direction-specific intercept of −82.37 °/s for rightward saccades. Converted EOG-derived velocities showed no significant differences from measured VOG-derived velocities. LOSO validation demonstrated stable transformation coefficients (mean slope = 0.147 °/μV, mean intercept = −82.47 °/s) and maintained agreement across individuals.

**Conclusion:**

A biophysically derived and experimentally validated EOG-to-VOG transformation model can provide accurate estimates of horizontal saccadic velocity under appropriate filtering conditions. These findings support the feasibility of quantitative saccadic analysis using EOG, providing a practical alternative to VOG.

## 1 Introduction

Saccadic eye movements, which are characterized by rapid shifts in the line of sight, serve as crucial biomarkers in various clinical and research applications (Bojrab et al., 2026). They comprise a hierarchy of behaviors, ranging from the most fundamental eye movements, such as the fast phases of vestibular nystagmus, to reactive saccades triggered by the sudden appearance of a novel target in the retinal periphery, and further to higher-level volitional behaviors, such as a series of saccades to scan the visual environment for new information (Munoz and Everling, 2004). These eye movements can be characterized by various parameters, including waveform, velocity, duration, trajectory, and accuracy, all of which can be used to estimate the integrity of the neural circuits from the cerebral cortex to the brainstem (McDowell et al., 2008; Saleh et al., 2015). Saccadic analysis is employed in many fields, including neuroscience, psychology, and the diagnosis and monitoring of neurological and psychiatric diseases (Gooding and Basso, 2008; Fracica et al., 2023).

Video-oculography (VOG) is an established technique for analyzing eye movements with high spatial and temporal resolutions (Baloh et al., 2010; Larrazabal et al., 2019). However, despite its widespread adoption, its high cost and resource-intensive setup pose challenges in many clinical environments (Stockwell, 1997; Mantokoudis et al., 2022). In addition, the limited portability of VOG systems restricts their use in settings where patient cooperation or stable visual access cannot be ensured (Leigh and Zee, 2015; Zhu et al., 2024). Electrooculography (EOG), primarily utilized in polysomnography (PSG) for sleep studies or electroencephalography (EEG), can reliably quantify eye movements by detecting corneo–retinal potential changes during eye movements (Toivanen et al., 2015; Markun and Sampat, 2020).

Advances in signal-processing techniques suggest that EOG can be optimized to provide reliable saccadic measurements, and its use has expanded from the detection of eye opening, closing, or gaze direction to more quantitative assessments of saccadic behavior (Barea et al., 2002; Dimigen et al., 2011; Belkhiria et al., 2022). Recent work has further shown that EOG signals can be processed to estimate ocular and gaze angles using model-based approaches, including methods that independently reconstruct the ocular position of each eye from EOG recordings (Barbara et al., 2025). Recent fusion algorithms grounded in a constant velocity model have been proposed to denoise EOG signals and improve the accuracy of EOG-based saccade parameter measurements while preserving key features of the saccadic waveform (Gunawardane et al., 2024). However, two important gaps remain in literature. First, previous studies have not systematically examined the relationship between EOG- and VOG-derived saccadic parameters, and the work addressing the quantitative correspondence between EOG measurements and VOG-equivalent values has so far been limited to specific experimental settings (Jia and Tyler, 2019; Pleshkov et al., 2022). Second, high-pass filter (HPF) design critically affects EOG waveform morphology; in particular, acausal filters can propagate later components backward in time and bias early signal segments, whereas causal filters introduce frequency-dependent attenuation that varies with cut-off frequency (Acunzo et al., 2012; Tanner et al., 2015; Widmann et al., 2015; Maess et al., 2016; Nunez et al., 2017). However, EOG–VOG comparison studies have not consistently evaluated the impact of filter settings on saccadic parameter estimation and the stability of EOG-to-VOG transformations (Terao et al., 2018; Jia and Tyler, 2019; Pleshkov et al., 2022).

Therefore, in this pilot feasibility study, we aimed to (1) investigate the correlation between EOG and VOG saccadic velocities, (2) propose a mathematically derived transformation model for converting EOG measurements into VOG-equivalent values, and (3) explore HPF conditions that may support stable EOG-based estimation of VOG-equivalent values. To the best of our knowledge, few studies have systematically investigated a mathematically derived EOG-to-VOG transformation approach together with the influence of HPF settings within the same experimental framework. Although preliminary and limited by the small sample size, this study provides proof-of-concept evidence supporting the potential feasibility of quantitative EOG-based saccadic analysis in situations where VOG may be impractical or unavailable.

## 2 Methods

### 2.1 Study population

This prospective observational study involved four healthy individuals (two female participants; age range: 33–44 years) with no history of neurological or sleep disorders, all of whom provided informed consent. Ethical approval for conducting experiments on human subjects was granted by Ajou University Hospital [Suwon, Republic of Korea, Institutional Review Board (IRB) no. AJOUIRB-IV-2025-253]. All testing sessions were conducted at the Ajou University Hospital in the Republic of Korea.

### 2.2 Data collection procedures

**Figure 1:**
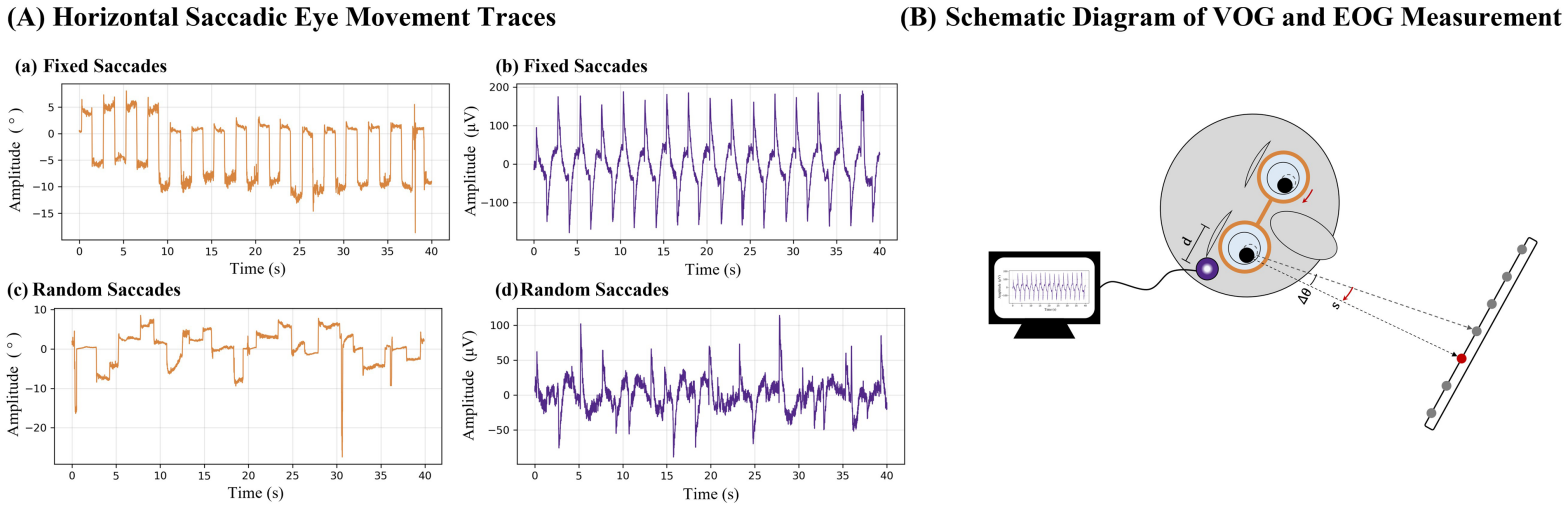
Horizontal saccadic eye movement traces and measurement schematic. **(A)** Horizontal saccadic eye movement traces of the right eye in a single subject (subject 1), recorded simultaneously using VOG (orange) and EOG (purple). The traces were time-synchronized to ensure accurate alignment between the two measurement modalities. **(B)** Schematic diagram of combined VOG and EOG measurement. The orange goggles represent the VOG device, while the purple circle indicates the location of the EOG electrode. The red dot represents the laser stimulus used to induce eye movement. d represents the initial distance between the electrode and the cornea when the eye is at rest. s refers to the corneal displacement caused by eye movement, and ∆𝜃 denotes the angle of the eye movement. The displacement s is calculated as s =𝛽·∆𝜃, where 𝛽 is a proportional constant. For ∆𝜃 ≤ 40°, the displacement s remains small enough to ensure 𝑑 > s.

Eye movements were recorded using EOG with the Comet-PLUS PSG system (Grass Technologies, Astro-Med, Inc., West Warwick, RI, USA) and using a VOG system (SLVNG, SLMED, Seoul, Republic of Korea). Calibration of the eye position was performed using a red dot sequentially presented at of ±10° from the central position in vertical and horizontal directions. During the procedure, the subjects were seated 1.2 m in front of the target (Kang and Kim, 2015). In the first experiment, the participants were instructed to shift the line of gaze to a visual target that appeared in the horizontal direction at an angle of 20° to the left and right, a task that was repeated every 2 s for a total of 15–16 trials. They were then instructed to shift their gaze to visual targets that appeared randomly in time and space within a ± 30° horizontal range, for 19–21 trials. Monocular horizontal eye movements were recorded from the right eye, with EOG signals sampled at 200 Hz and VOG signals sampled at 120 Hz. Representative horizontal saccadic eye movements traces from both systems are shown in Figure 1A. The experimental setup, including the relative positions of the subject, visual targets, and recording systems, is schematically illustrated in Figure 1B. Both the EOG and VOG signals were recorded simultaneously, resulting in two parallel data streams during the two sessions. Each recording process lasted for 40 s, and all tests were performed by an instructed physician. Saccadic transition points in the VOG traces produced in response to red-dot visual targets were visually identified by a neuro-ophthalmology specialist (S.-U. L.). Time synchronization between the systems was achieved by matching the red dot onset recorded in the VOG timeline with the corresponding red dot cues in the EOG records. This procedure enabled accurate time synchronization between the different sampling rates. VOG recordings were exported as *.emd* files and preprocessed using MATLAB (R2023b, MathWorks Inc., Natick, MA, USA), whereas the EOG recordings were exported as *.edf* files and analyzed using Python (v3.11.5; Python Software Foundation, Wilmington, DE, USA) with NumPy (v1.24.4), MNE (v1.6.0), and SciPy (v1.11.1).

### 2.3 Signal processing and filtering

The EOG signal was preprocessed using causal digital filters to examine the influence of the cut-off frequency on the signal morphology and EOG saccadic velocity estimation. Waveform distortion across different filter conditions was evaluated by generating and analyzing synthetic EOG waveform (−20 to +20 μV) simulating alternating rightward and leftward saccades at multiple HPF cut-off frequencies (0.1, 0.3, and 1 Hz). In addition, multiple HPF conditions were applied to the recorded EOG signals to systematically assess the influence of each filtering cut-off frequency on the stability of the subsequent transformation models relating the EOG and VOG saccadic velocities. To isolate the effect of HPF settings, the low-pass filter (LPF) cut-off frequency was fixed at 35 Hz throughout all analyses according to previously reported technical recommendations for EOG signal recordings (Peltola et al., 2023). Synthetic waveform analyses showed that increasing the HPF cut-off frequency progressively altered the shape of the simulated saccadic waveform and affected both amplitude and velocity estimates. By contrast, the LPF primarily attenuated high-frequency noise, such as muscle-related artifacts, and had relatively little influence on overall waveform morphology and velocity estimation. Therefore, only the HPF cut-off frequency was systematically varied, while the LPF setting was kept constant to minimize additional variability and allow a focused evaluation of HPF-related effects on EOG-to-VOG transformation performance. In addition to such conventional high-pass filtering strategies, model-based baseline drift mitigation techniques have been proposed that exploit target gaze angles within a battery model of the eye to estimate and remove the baseline component of the EOG signal while preserving its morphology (Barbara et al., 2021). In the present pilot study, however, we focused specifically on varying the HPF cut-off frequency while keeping the LPF constant at 35 Hz, in order to isolate HPF-related effects on waveform shape and the stability of the EOG-to-VOG transformation.

### 2.4 Saccadic velocity calculation

VOG saccadic velocity (𝑣_𝑣_): 𝑣_𝑣_ is defined as the angular velocity of the eye movement, calculated by dividing the change in gaze position (angular displacement *Δ*𝜃, expressed in degrees (°)) by the time taken to complete the movement (Δ𝑡):


$$v_{v}=\frac{\Delta \theta }{\mathrm{\Delta t}}\;\;\;{(}^{{}^{\circ}}/s)$$ (1)

EOG saccadic velocity (𝑣*_e_*): 𝑣*_e_* is defined as the rate of change in the recorded EOG signal amplitude (Δ𝑉), normalized over time:


$$v_{e}=\frac{\mathrm{\Delta V}}{\mathrm{\Delta t}}\;\;\;(\mathrm{\mu V}/s)$$ (2)

### 2.5 Statistical analysis

Linear regression analysis was used to derive transformation equations between EOG-derived saccadic velocity (*v_e_*) and VOG-derived saccadic velocity (*v_v_*). Based on the theoretical assumption that zero EOG velocity corresponds to zero VOG velocity under ideal conditions, leftward saccades were modeled using a regression constrained to pass through the origin (*v_v_ = kv_e_*). For rightward saccades, an intercept term was additionally evaluated to account for potential direction-dependent asymmetry, and a regression model including both slope and intercept terms (*v_v_ = kv_e_ + b*) was examined. The statistical significance of the estimated slope and intercept coefficients was assessed using regression coefficient test, with *p*-values reported for each parameter.

To evaluate the agreement between converted EOG velocities and corresponding VOG velocities, paired t-test and Bland–Altman analyses were performed. Paired t-tests were used to determine whether significant differences existed between the two parameters. Bland–Altman analysis was used to assess systematic bias and agreement between methods by calculating the mean bias, lower and upper limits of agreement (LoA), and LoA width. In addition, mean absolute error (MAE) and root mean square error (RMSE) were calculated as measures of prediction error. A p-value < 0.05 was considered statistically significant.

To assess the generalizability of the transformation model across individuals, leave-one-subject out (LOSO) validation was performed. In each iteration, one participant was excluded from model derivation and used as an independent test subject, while the remaining participants were used to estimate the transformation model.

## 3 Results

### 3.1 Acquisition of saccadic velocity range

Table 1 summarizes the saccadic velocities of the right eye measured using VOG and EOG systems across all recorded horizontal saccades pooled from four participants (total number of saccades = 151). The VOG system recorded a mean rightward saccadic velocity of 193.5 ± 62.3°/s, whereas leftward movements showed a mean velocity of −203.3 ± 83.3°/s. In contrast, the EOG system recorded a mean rightward saccadic velocity of 1891.0 ± 464.7 μV/s and a mean leftward velocity of −1345.5 ± 333.3 μV/s. The VOG system recorded slightly higher absolute velocities for leftward than rightward saccades, whereas the EOG system demonstrated a more pronounced directional asymmetry, with substantially larger absolute values during rightward movements. This directional differences in EOG recordings motivated the use of direction-specific transformation models in subsequent analyses.

**Table 1:** Comparison of saccadic velocities of the right eye between VOG, EOG and converted EOG.

		4 Subjects			*p*-value
		VOG-derived velocity (°/s)	EOG-derived velocity (μV/s)	Converted EOG-derived velocity (°/s)	
Horizontal saccadic velocity (*n* = 151)	Rightward	193.5 ± 62.3	1891.0 ± 464.7	193.5 ± 67.8	1.000
	Leftward	− 203.3 ± 83.3	− 1345.5 ± 333.3	− 196.3 ± 48.7	0.479

Saccadic velocities are shown for all recorded saccades pooled from four subjects (*n* = 151). Values are presented as mean ± standard deviation (SD). Statistical comparisons between VOG-derived and converted EOG-derived velocities were conducted using paired t-tests, and the resulting *p*-values are reported to indicate statistical significance.

### 3.2 Development of the proposed transformation model

Figure 1B shows a schematic of the EOG measurement from an electrode placed lateral to one eye. The relationship between corneal displacement (*s*), eye movement angle (*Δθ*), and voltage changes was modeled as follows:

① Biophysical model of the eyeball

The eyeball was simplified as an electrical source, with the cornea modeled as an effective point source and the retina as a negative pole forming a dipole structure. Given the proximity of the EOG electrode to the eyeball, a near-field model was adopted instead of a far-field dipole approximation. Accordingly, the electric potential measured at the EOG electrode was described based on the current-source analog of Coulomb’s law, corresponding to the electric potential generated by a point current source in a homogeneous volume conductor (Equation 3):


$$V=\frac{I}{4\mathrm{\pi \sigma r}}$$ (3)

where *σ* denotes the conductivity of the medium, *I* the effective source strength or current, and *r* the distance from the source to the electrode. In the present model, the corneal contribution was represented as an effective point source with strength *I_c_*, while the retinal contribution was represented as a sink with strength *I_r_*. Accordingly, the voltage measured at the EOG electrode can be expressed as:


$$V=\frac{1}{4\pi }(\frac{I_{c}}{\sigma_{c}r_{c}}-\frac{I_{r}}{\sigma_{r}r_{r}})$$ (4)

where *σ_c_* and *σ_r_* denote the effective conductivities of the surrounding tissue from the cornea and retina to the electrode, respectively, and *r_c_* and *r_r_* represent the corresponding distances.

② Cornea-dominant approximation

Due to anatomical and volume conductor properties, the effective conductivity differs between the corneal and retinal pathways. The corneal signal propagates through relatively conductive superficial tissues, whereas the retinal signal traverses deeper structures with lower conductivity. Consequently, the retinal contribution is substantially attenuated compared to the corneal contribution. Based on this consideration, Equation 4 can be approximated by the cornea-dominant model (Equation 5):


$$V\approx \frac{I_{c}}{4\pi \sigma_{c}r_{c}}$$ (5)

This approximation assumes that *σ_r_* ≪ *σ_c_*, leading to a negligible contribution of the retinal term to the measured potential.

③ Geometric representation of eye movement

For horizontal eye movement, the corneal position follows a circular trajectory with radius *a*, and the arc displacement is defined as:


$$s=\mathrm{a\Delta}\theta_{\mathrm{rad}}=a\frac{\pi }{180}\Delta \theta$$ (6)

where *Δθ* is the eye rotation angle in degree and *Δθ_rad_* in radians.

④ Analytical approximation of voltage changes and small eye movements

The distance *d* is defined as the separation between the corneal center and the EOG electrode when the eye is in the primary position (*θ* = 0). Assuming that the corneal displacement is small relative to the electrode distance (i.e., *s* ≪ *d*), the voltage difference between positions +*s*/2 and −*s*/2, corresponding to angular positions +*Δθ*/2 and −*Δθ*/2, is given by:


$$\mathrm{\Delta V}\approx \frac{I_{c}}{4\pi \sigma_{c}}\{\frac{1}{\left(d-s/2\right)}-\frac{1}{\left(d+s/2\right)}\}=\frac{I_{c}}{4\pi \sigma_{c}}\{\frac{s}{\left(d^{2}-s^{2}/4\right)}\}$$ (7)

Under the small-displacement assumption (*s* ≪ *d*) and using Equation 6, Equation 7 simplifies to:


$$\mathrm{\Delta V}\approx \frac{I_{c}}{4\pi \sigma_{c}}\frac{s}{d^{2}}=\frac{aI_{c}}{720d^{2}\sigma_{c}}\Delta \theta$$ (8)

Rearranging Equation 8 gives:


$$\Delta \theta \approx \frac{720d^{2}\sigma_{c}}{aI_{c}}\mathrm{\Delta V}=\mathrm{k\Delta V}$$ (9)

where *k* (=720*d^2^σ_c_/aI_c_*) is a constant.

⑤ Final relationship Between EOG and VOG velocities

Taking the time derivative of Equation 9 yields Equation 10:


$$\frac{\Delta \theta }{\mathrm{\Delta t}}\approx k\frac{\mathrm{\Delta V}}{\mathrm{\Delta t}}$$ (10)

Using Equations 1, 2, the relationship between EOG and VOG velocities is:


$$v_{v}\approx k\times v_{e}$$ (11)

Thus, the voltage velocity is linearly proportional to the angular eye velocity under small-angle conditions.

⑥ Numerical simulation of the linear approximation

To validate the analytical approximation, numerical simulations were conducted without applying the simplifying assumptions used in the theoretical derivation, as detailed in the Supplementary materials. The linear approximation (Equation 11) was evaluated against the full corneo-retinal dipole model (Equation 4). The results demonstrated that the linear approximation remained valid when compared with the full corneo-retinal dipole simulation incorporating realistic EOG electrode positioning and physiological eye geometry. Across saccade amplitudes ranging from ±5° to ±45°, the estimated saccadic *dV*/*dt* values agreed within 4% of those obtained from the full model (Supplementary Methods and Results, Supplementary Figure S1).

⑦ Simulation of direction-dependent velocity gain

To investigate whether direction-dependent differences in the EOG-to-VOG transformation could arise from asymmetries in signal formation and processing, an additional numerical simulation was performed. Symmetric horizontal saccades with identical amplitudes, durations, and overshoot characteristics were generated using a minimum-jerk trajectory model, and EOG signals were synthesized using a nonlinear voltage-mapping function incorporating direction-dependent gain factors, saturation effects, and a small asymmetry term representing differences in effective electrode sensitivity and lead-field geometry (Bahill et al., 1975). After application of a first-order high-pass filter (0.3 Hz), only a minimal difference in velocity gain was observed between directions, with the estimated transformation coefficient differing by approximately 0.4%. This difference was considered negligible relative to the variability observed in the experimental data; therefore, the transformation coefficient (*k*) considered theoretically equivalent for both directions, supporting the use of a common slope coefficient in the final transformation model.

⑧ Simulation of direction-dependent velocity gain

In the derivation of the preceding equations, the electrical contributions of the extraocular muscles were not explicitly considered. Such muscle-related activity may influence the recorded EOG signal independently of the corneo-retinal potential (Keren et al., 2010), and may therefore contribute to both the velocity-dependent variance term (*ε*) and the velocity-independent offset term (*b*). Accordingly, Equation 11 may be reformulated as:


$$v_{v}\approx k\times v_{e}+\varepsilon \times v_{e}+b$$ (12)

Theoretically, the coefficient (*k*) should be identical for symmetrical leftward and rightward saccades. However, directional asymmetry may arise from differences in the anatomical relationship between the recording electrode and the extraocular muscles. During a rightward saccade of the right eye, the lateral rectus muscle is activated in close proximity to the lateral EOG electrode, whereas the medial rectus muscle activated during leftward saccades is located relatively farther away. Electrical activity associated with the lateral rectus muscle may therefore contribute more strongly to the measured EOG signal. Consequently, the practical EOG-to-VOG relationship may deviate from the idealized model in a direction-dependent manner.

Under this framework, the leftward transformation may be approximated as:


$$v_{v_{\text{left}}}\approx k\times v_{e_{\text{left}}}$$ (13)

whereas the rightward transformation may be represented as:


$$v_{v_{\text{right}}}\approx k\times v_{e_{\text{right}}}+\varepsilon_{\text{right}}\times v_{e_{\text{right}}}+b_{\text{right}}$$ (14)

### 3.3 Filtering analysis and selection

**Figure 2:**
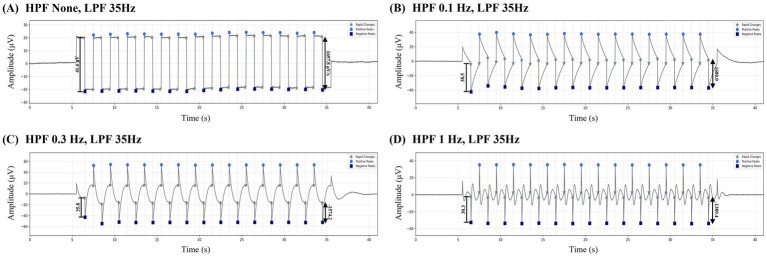
Comparison of saccadic velocities under high-pass filter (HPF) cut-offs on signal waveform in a synthetic EOG. **(A)** HPF None, LPF 35 Hz. **(B)** HPF 0.1 Hz, LPF 35 Hz. **(C)** HPF 0.3 Hz, LPF 35 Hz. **(D)** HPF 1 Hz, LPF 35 Hz. Rapid change points denote saccade onset. Positive peaks correspond to right-gaze fixation, whereas negative peaks correspond to left-gaze fixation. Saccadic velocity represents the voltage change from onset to each fixation target. All synthetic EOG waveforms were processed using a fixed 35 Hz low-pass filter (LPF). Gaussian noise and baseline drift were added to emulate real-world EOG characteristics.

Synthetic EOG signals were generated to evaluate the HPF-induced waveform distortion and saccadic velocity changes at various filter settings. The resulting waveform morphologies and changes in saccadic velocities were compared across the filtering conditions (Figure 2). The synthetic EOG traces demonstrated progressive waveform distortion as the HPF cut-off frequency increased. Consistent with the experimental recordings, increasing the HPF cut-off frequency resulted in progressively lower EOG-derived saccadic velocities, showing generally consistent changes in both synthetic and real-world EOG signals (Supplementary Table S1).

Based on these preliminary observations, multiple HPF conditions were applied to the recorded EOG signals, and transformation models were estimated using the pooled dataset of all recorded horizontal saccades. Direction-specific transformation coefficients were derived for each HPF setting, and the resulting relationships between EOG-derived and VOG-derived saccadic velocities were examined (Supplementary Figure S2). For leftward saccades, statistically significant regression slope were observed under all HPF conditions (all *p* < 0.001). For rightward saccades, the estimated intercept remained relatively stable across the filtering conditions, ranging from −70.965 to −82.370^°^/s.

**Figure 3:**
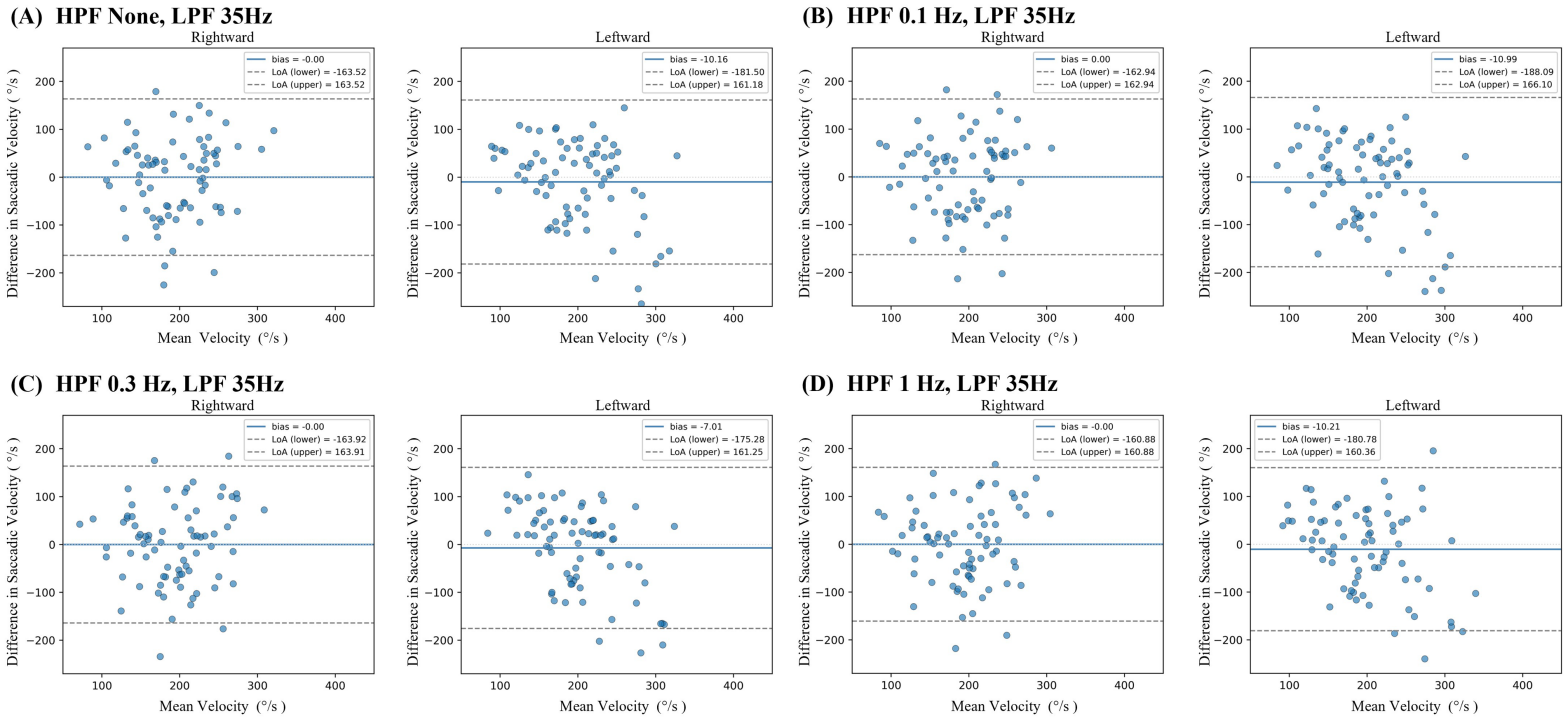
Bland-Altman plots showing agreement between converted EOG-derived and VOG-derived saccadic velocities across different HPF conditions. **(A)** HPF None, LPF 35 Hz. **(B)** HPF 0.1 Hz, LPF 35 Hz. **(C)** HPF 0.3 Hz, LPF 35 Hz. **(D)** HPF 1 Hz, LPF 35 Hz. For each HPF condition, rightward and leftward saccades are present separately. In each Bland-Altman plot, the x-axis represents the mean of the converted EOG-derived and VOG-derived saccadic velocities, and the y-axis represents the difference between the converted EOG-derived and VOG-derived saccadic velocities (converted EOG – VOG). The solid blue line indicates the mean difference (bias), and the dashed gray lines indicate the 95% limits of agreement (LoA).

No significant differences were observed between the VOG-derived and converted EOG-derived velocities under any HPF condition (all paired t-test *p* > 0.05; Supplementary Table S2). Bland–Altman analysis further demonstrated comparable agreement across filter settings (Figure 3). When all recorded saccades were pooled, the 0.3 Hz HPF condition yielded the lowest mean bias (3.53^°^/s), MAE (67.97^°^/s), and RMSE (84.34^°^/s). Direction-specific analysis additionally showed that the 0.3 Hz HPF condition yielded the best overall performance for leftward saccades, with the lowest bias (7.01^°^/s), LoA width (336.53^°^/s), MAE (68.70^°^/s), and RMSE (85.57^°^/s), whereas only marginal improvements were observed for rightward saccades at 1 Hz. Taken together, these findings supported the selection of the 0.3 Hz HPF condition for the final transformation model.

### 3.4 Transformation model derivation

Under the selected filtering condition (0.3 Hz HPF, 35 Hz LPF), direction-specific transformation equations were derived between EOG saccadic velocity (𝜇𝑉/𝑠) and VOG saccadic velocity (°/s) for both rightward and leftward saccades of the right eye (Supplementary Figure S2).

- Leftward saccade ( − direction): The regression Equation 13 was

$$v_{v_{\text{left}}}=0.146\times v_{e_{\text{left}}}$$

- Rightward saccade ( + direction): The regression Equation 14 was

$$v_{v_{\text{right}}}=0.146\times v_{e_{\text{right}}}-82.370$$

To determine the practical form of the transformation model for rightward saccade, the variance-related term (*ε_right_*) and offset term (*b_right_*) introduced in Equation 14 were evaluated using experimental data. The variance-related component did not provide a meaningful improvement in model fit and was therefore omitted from the final model, whereas the offset term remained significant for rightward saccades. Accordingly, the final transformation equations were derived using a common slope coefficient with a direction-dependent intercept term (Supplementary Figure S1C). The intercept term was statistically significant (*p* = 0.003) whereas the leftward model was adequately described by a regression constrained to pass through the origin. The estimated slope was statistically significant (*p* < 0.001).

### 3.5 Comparison of VOG velocity and converted EOG velocity

After application of the final transformation model (0.3 Hz HPF, 35 Hz LPF), no significant differences were observed between the converted EOG-derived and VOG-derived saccadic velocities for either rightward or leftward saccades (Table 1). The converted EOG-derived velocities closely approximated the corresponding VOG-derived velocities, with mean values of 193.5 ± 67.8°/s vs. 193.5 ± 62.3°/s for rightward saccades and −196.3 ± 48.7°/s vs. −203.3 ± 83.3°/s for leftward saccades.

**Figure 4:**
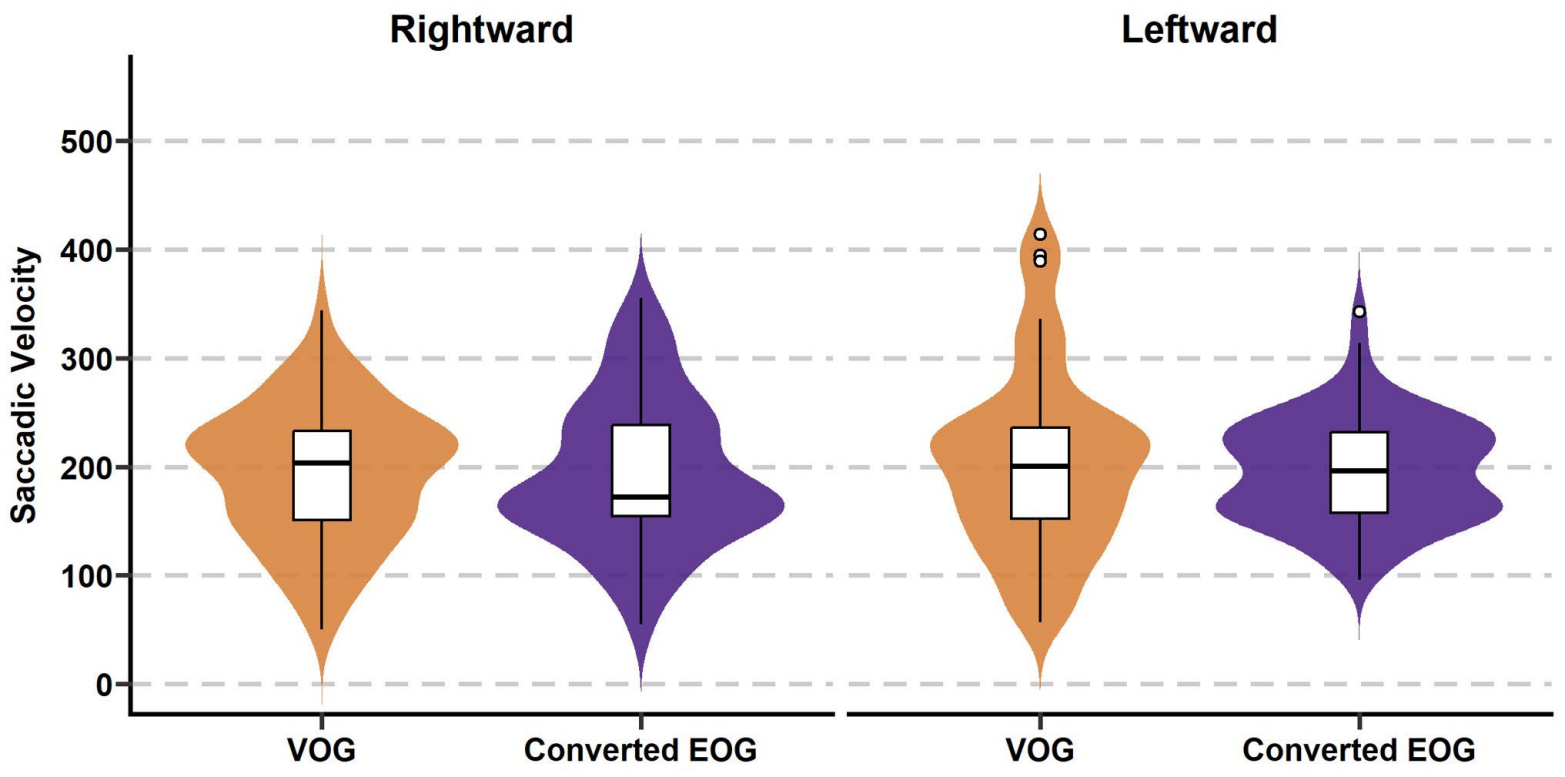
Violin plots comparing saccadic velocities of the right eye between VOG and converted EOG (using transformation model). Each violin plot shows the full distribution of velocities; central white box = interquartile range (IQR), horizontal line = median, and outer shapes = probability density.

Figure 4 illustrates the distributions of VOG-derived and converted EOG-derived saccadic velocities. The overall distribution showed substantial overlap for both movement directions, indicating that the transformation model preserved the central tendency and distributional characteristics of the original VOG measurements. Comparable distributional patterns were also observed across the remaining HPF conditions, as shown in Supplementary Figure S3.

To evaluate the generalizability of the transformation model across individuals, leave-one-subject-out (LOSO) validation was performed (Supplementary Table S3). Across the four validation iterations, the estimated transformation coefficients remained relatively stable (mean slope = 0.147, mean intercept = −82.467). Agreement metrics obtained from the held-out participants showed a mean bias of 3.99°/s, LoA width of 372.70°/s, MAE 78.31°/s, and RMSE of 94.14°/s. Although prediction errors were moderately larger than those obtained using the model derived from the pooled dataset (bias = 3.53°/s, LoA width = 331.42°/s, MAE = 67.97°/s, RMSE = 84.34°/s), the overall agreement remained comparable, supporting the feasibility of applying the proposed transformation approach to previously unseen individuals.

## 4 Discussion

### 4.1 Significance of the study

This pilot feasibility study provides preliminary evidence supporting the use of EOG for estimating VOG-equivalent saccadic velocities. By examining the relationship between EOG-derived and VOG-derived average saccadic velocities, we demonstrated that a simple transformation approach can reduce the discrepancy between the two measurement modalities and produce comparable velocity estimates under selected filtering conditions. Although further validation is required, these findings suggest that EOG may have potential as a practical alternative for quantitative saccadic assessment in situations where VOG measurements are difficult to obtain.

In clinical settings, VOG is typically performed in cooperative patients and requires continuous visualization of the pupil. Because EOG can be recorded simultaneously with EEG or PSG and does not depend on direct visualization of the eye, it may offer a complementary approach for evaluating eye movements in these settings. Therefore, the present findings provide preliminary support for further investigation of EOG-based quantitative eye movement assessment in both clinical and research applications.

### 4.2 Saccadic velocity comparison

Prior studies have reported that the average horizontal peak saccadic velocities exceed 200°/s for 30° saccades in normal subjects (Yu et al., 2018). Although average rather than peak velocity was analyzed in the present study, the measured values were comparable to those reported previously and remained within the expected physiological range. Because average and peak velocities are closely related through the main-sequence relationship, the use of average velocity is unlikely to substantially affect the validity of the proposed transformation model.

### 4.3 Agreement between converted EOG-derived and VOG-derived velocities

The present findings suggest that EOG-derived saccadic velocities can be transformed to approximate corresponding VOG-derived measurements under the experimental conditions examined in this study. Following application of the transformation equations, the converted EOG velocities demonstrated a level of agreement with VOG velocities that was maintained across multiple complementary analyses, including paired comparisons, Bland–Altman analysis, and distribution-based visualization. Together, these findings support the feasibility of estimating VOG-derived saccadic velocities from EOG recordings using a simple regression-based approach.

An important observation was that the transformation model was able to preserve the overall distributional characteristics of the VOG measurements despite the directional asymmetry present in the raw EOG recordings. This suggests that much of the discrepancy between the two modalities may be attributable to systematic measurement-related factors rather than fundamental differences in the underlying saccadic behavior. Consequently, the proposed transformation approach may provide a practical framework for reducing modality-dependent differences when quantitative saccadic velocity measurements are required.

Beyond such regression-based transformations, model-based fusion techniques that combine lumped-element eye models with Kalman filtering have also been used to enhance saccade-related features in noisy EOG signals and improve EOG-based saccade parameter estimation compared with conventional band-pass filtering (Gunawardane et al., 2021). Taken together, these model-based enhancement methods and the present transformation framework may provide complementary tools for obtaining VOG-equivalent saccadic velocities from EOG recordings.

The LOSO analysis provided an additional exploratory assessment of model performance across individuals. Although agreement metrics were modestly reduced when the model was applied to held-out participants, performance remained broadly comparable to that observed in the pooled dataset. These findings suggest that the transformation approach may retain utility beyond the specific individuals used for model derivation.

### 4.4 Directional asymmetry in transformation constants

Although the initial linear theoretical model (Equation 11) predicts an identical transformation equation for rightward and leftward saccades, the experimental data demonstrated a systematic directional bias. Rather than introducing separate transformation coefficients for each direction, we modeled this asymmetry using a common coefficient together with an additional offset term (Equation 12). This approach was based on the assumption that the dominant source of directional asymmetry arises from additive physiological artifacts rather than from fundamental differences in the voltage-to-velocity relationship itself.

A plausible explanation for this directional bias is the influence of extraocular muscle activity (Keren et al., 2010), particularly from the lateral rectus muscle, which is anatomically closer to the recording electrode than the medial rectus muscle. Such activity may introduce a direction-specific offset, leading to a systematic difference between rightward and leftward saccades despite an otherwise similar transformation coefficient.

Other factors may also contribute to the observed asymmetry. Variations in electrode placement, bipolar channel configuration, and dipole-to-electrode geometry can alter the effective sensitivity of the recording system (Pleshkov et al., 2022; Barbara et al., 2026). In addition, volume conduction through biological tissues, including anisotropic conductivity of the skull and surrounding structures, may modify the spatial distribution of electrical potentials (Vorwerk et al., 2014). Recording system characteristics such as high-pass filtering, amplifier phase response, and electrode–skin impedance may further affect signal amplitude and waveform morphology. Moreover, VOG-related factors, including tracking inaccuracies and exclusion of unreliable signal segments, may influence the comparison between EOG- and VOG-derived velocity measures.

Finally, physiological factors such as translational eye movements, eyelid motion, skin deformation, torsional eye movements, and intrinsic directional asymmetries of saccadic dynamics may also contribute (Vergilino-Perez et al., 2012). Therefore, while the additional offset term is most likely attributable to lateral rectus muscle activity, other measurement-related and physiological factors cannot be completely excluded. Future studies incorporating dedicated EMG recordings or refined biophysical models may help quantify the relative contribution of these factors.

### 4.5 Clinical and research implications

VOG is widely regarded as the reference standard for quantitative eye movement analysis because it directly measures eye position and velocity with high spatial and temporal resolution (Baloh et al., 2010). However, VOG relies on continuous visualization of the pupil and therefore may be difficult to apply in patients who have impaired eyelid opening or are being monitored during sleep (Leigh and Zee, 2015).

In contrast, EOG does not require direct visualization of the eye and can be recorded simultaneously with EEG or PSG using relatively simple instrumentation (Young and Sheena, 1975). The present findings suggest that EOG-derived saccadic velocities may be quantitatively related to corresponding VOG-derived measurements under controlled experimental conditions. Therefore, EOG-based recordings may provide complementary information for quantitative eye movement assessment in situations where conventional video-based eye tracking is difficult to perform or unavailable. Although the present findings do not establish clinical applicability, they support further investigation of EOG-based quantitative eye movement analysis in both clinical and research settings.

### 4.6 Limitations and future directions

Several limitations of this pilot feasibility study should be considered when interpreting the present findings. First, the study included only four healthy participants, and the transformation equations were derived from a relatively small dataset. Although exploratory LOSO validation suggested that the overall pattern of agreement was maintained across individuals, the current results should not be interpreted as establishing full generalizability of the proposed transformation approach. Furthermore, because all participants were healthy individuals, it remains unclear whether the proposed transformation would perform similarly in patients with neurological, ophthalmological, or vestibular disorders that affect saccadic eye movements. Second, the present study was also limited to monocular recordings from the right eye during controlled horizontal saccades; therefore, whether similar relationships exist in the left eye or during more complex eye movement behaviors, including free-viewing tasks, smooth pursuit, and other naturalistic visual conditions, remains to be determined. Furthermore, the proposed model was derived using only symmetric saccades centered on the primary gaze position. Future studies involving larger and more diverse populations, bilateral recordings, and broader eye movement paradigms will be necessary to evaluate the reproducibility, stability, and clinical applicability of the proposed transformation approach. Finally, all velocity measures represent average saccadic velocity over the entire movement duration rather than instantaneous peak velocity. Given the sampling rates of the VOG (125 Hz) and EOG (200 Hz) systems, average velocity was considered more robust and practical for the proposed transformation. Future studies with higher temporal-resolution recordings are needed to validate the proposed transformation model using peak saccadic velocity.

## 5 Conclusion

This pilot feasibility study examined the quantitative relationship between EOG-derived and VOG-derived saccadic velocities and proposed a simple transformation approach for estimating VOG-derived measurements from EOG recordings. Under the selected filtering condition, the transformed EOG-derived velocities showed agreement with the corresponding VOG-derived velocities across multiple complementary analyses. These findings suggest that EOG recordings may contain quantitative information relevant to saccadic velocity assessment and support continued investigation of EOG-based quantitative eye movement analysis in clinical and research settings.

## Data Availability

The raw EOG and VOG recordings are not publicly available because of institutional and ethical restrictions. Processed data supporting the findings of this study, including the derived quantitative measurements used for statistical analyses, may be made available by the corresponding author upon reasonable request, subject to approval by the authors and compliance with applicable institutional and ethical requirements.
