# Supplementary material for: Development of a transformation model to analyze horizontal saccadic velocity using electrooculography: a pilot feasibility study
Source: Front Neurosci. 2026 Jul 7;20:1862069. doi: 10.3389/fnins.2026.1862069 (PMC13384945; doi:10.3389/fnins.2026.1862069)
Supplement: Supplementary file 1 [file Data_Sheet_1.docx]

Supplementary Material

# Supplementary Methods, Results and References

## Supplementary Methods

To validate the analytical approximation, numerical simulations were performed without applying the simplifying assumptions used to derive the linear relationship between EOG and VOG velocities in the Results section. In the main analysis, linearity was obtained based on three key approximations; however, to assess whether this relationship holds under more realistic nonlinear conditions, simulations were conducted using the full nonlinear formulation without these assumptions.

Specifically, the following conditions were implemented without approximation: (1) the retinal contribution was retained with a reduced effective conductivity relative to the corneal pathway, (2) the exact geometric relationship was used without enforcing the small-displacement assumption (s ≪ d), and (3) realistic electrode positioning was incorporated based on anatomical considerations.

The right EOG electrode was positioned approximately 1 cm lateral and 1 cm superior to the right lateral canthus, consistent with standard electrode placement. Considering that the horizontal palpebral fissure length in adults is approximately 2.8–3.0 cm, the lateral component of the position vector was set to r_x_ ≈ 2.5 cm. In addition, given that the eyeball depth is approximately 2.2–2.4 cm and that the lateral orbital wall exhibits a posterior curvature formed by the zygomatic bone and temporalis muscle, the electrode is effectively positioned slightly posterior relative to the lateral canthus while the eyeball protrudes anteriorly. Based on these anatomical considerations, the anterior offset was approximated as r_y_ ≈ 0.5 cm. The vertical component was set to r_z_ ≈ 1.0 cm according to electrode placement. Accordingly, the electrode position vector relative to the eyeball center was approximated as:

$$\mathbf{r}=\left[ r_{x}, r_{y}, r_{z} \right]\approx\left[ 2.5, 0.5, 1.0 \right] \mathrm{cm}$$

In addition, the temporal profile of corneal angular movement was modeled based on previously reported characteristics of saccadic eye movements (1). Specifically, the angular velocity rapidly increased following movement onset, reached a peak velocity, and then symmetrically decreased. The peak angular velocity was assumed to follow a logarithmic relationship with movement amplitude, consistent with prior studies.

## Supplementary Results

To assess the adequacy of the linear approximation (equation (9)) relative to the more physically realistic cornea-retina dipole model (equation (4)) for describing the EOG signal generated by horizontal saccades, we simulated saccades over a range of amplitudes using a triangular angular-velocity profile and the main-sequence relation *ω_peak* = 95·(*Δθ*)^0.5 deg/s. For each simulated saccade, the instantaneous and the saccade rates of change of recorded voltage (*dV/dt*) were computed using both voltage models, with the conductivity ratio set to *κ* = *σ_r_* / *σ_c_* = 10 in the dipole model.

By construction, the linear approximation yields an instantaneous *dV*/*dt* that is exactly proportional to the angular eye velocity *dθ*/*dt*, with a constant scaling factor *C*(equation (8)); the two waveforms therefore have identical shape and differ only in their vertical scale (Supplementary Figure S1A). The cornea-retina dipole model, owing to the geometric nonlinearity in the inverse-distance terms of equation (4), produces a slightly different instantaneous waveform. As shown in Supplementary Figure 1, this difference in shape between the dipole prediction and the linear approximation becomes more pronounced as saccade amplitude increases. Despite these differences in waveform shape, however, the peak values of instantaneous *dV*/*dt* were not substantially different between the two models, and neither were the saccadic values. When the saccadic *dV*/*dt* alone was compared (Supplementary Figure S1B), the dipole simulation and the linear approximation agreed to within 4 % across the entire range of amplitudes tested (±5° to ±45°). Furthermore, the linear approximation produced a relationship between dV/dt and dθ/dt that was itself nearly perfectly linear through the origin. Taken together, these results indicate that the linear approximation provides an adequate description of the EOG voltage rate produced during saccades over a wide range of amplitudes, even though the underlying dipole geometry introduces small but systematic nonlinearities in the instantaneous waveform that grow with saccade amplitude.

## Supplementary References

1. Bahill, A. T., Clark, M. R., and Stark, L. (1975). The main sequence, a tool for studying human eye movements. Math. Biosci. 24, 191–204.

# Supplementary Figures and Tables

##
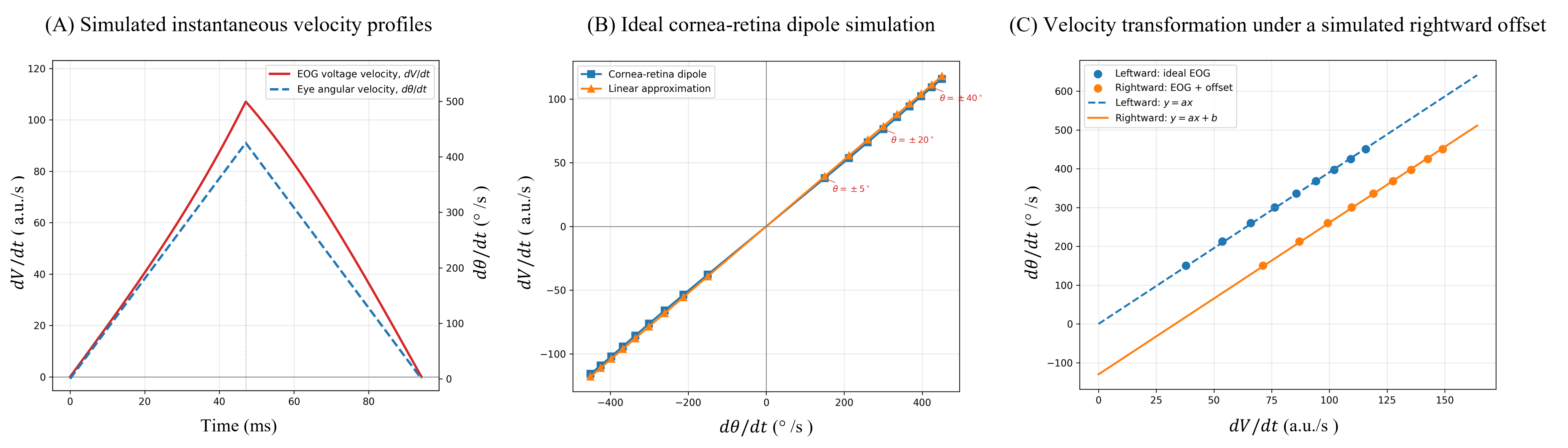
Supplementary Figures

**Supplementary Figure S1.** Simulated relationship between eye angular velocity and EOG voltage velocity under an ideal cornea-retina dipole model and conceptual effect of a direction-specific offset. (A) Simulation performed under an ideal cornea-retina dipole model without extraocular muscle activity or other physiological artifacts. Instantaneous EOG voltage velocity (𝑑𝑉/𝑑𝑡) and eye angular velocity (𝑑𝜃/𝑑𝑡) were calculated during a representative horizontal saccade (-10° to +10°). The EOG voltage velocity closely follows the temporal profile of the eye angular velocity throughout the movement, supporting a proportional relationship between the two quantities under ideal conditions. (B) Simulation performed under the same cornea-retinal dipole model. EOG voltage velocity ($dV/dt)$ was plotted against eye angular velocity ($d\theta/dt$) for both rightward and leftward horizontal saccades. The two directions lie on the same linear relationship ($y=ax$), indicating that the transformation slope is theoretically identical for rightward and leftward movements under ideal conditions. The linear approximation closely overlaps the full dipole model, demonstrating that this proportional relationship is preserved under a first-order approximation. (C) Conceptual simulation illustrating the effect of a direction-specific additive component on the velocity transformation. Using the common slope predicted by the ideal cornea-retina dipole model, a constant offset was added to the simulated rightward EOG voltage velocity while preserving the original transformation slope. Under this condition, rightward and leftward movements remain characterized by a common slope ($a$), but require different intercept terms ($y=ax$ and $y=ax+b$). This simulation demonstrates how a direction-dependent additive component, such as a local physiological signal unrelated to eye rotation, could produce asymmetric transformation equations despite an identical underlying slope.

**
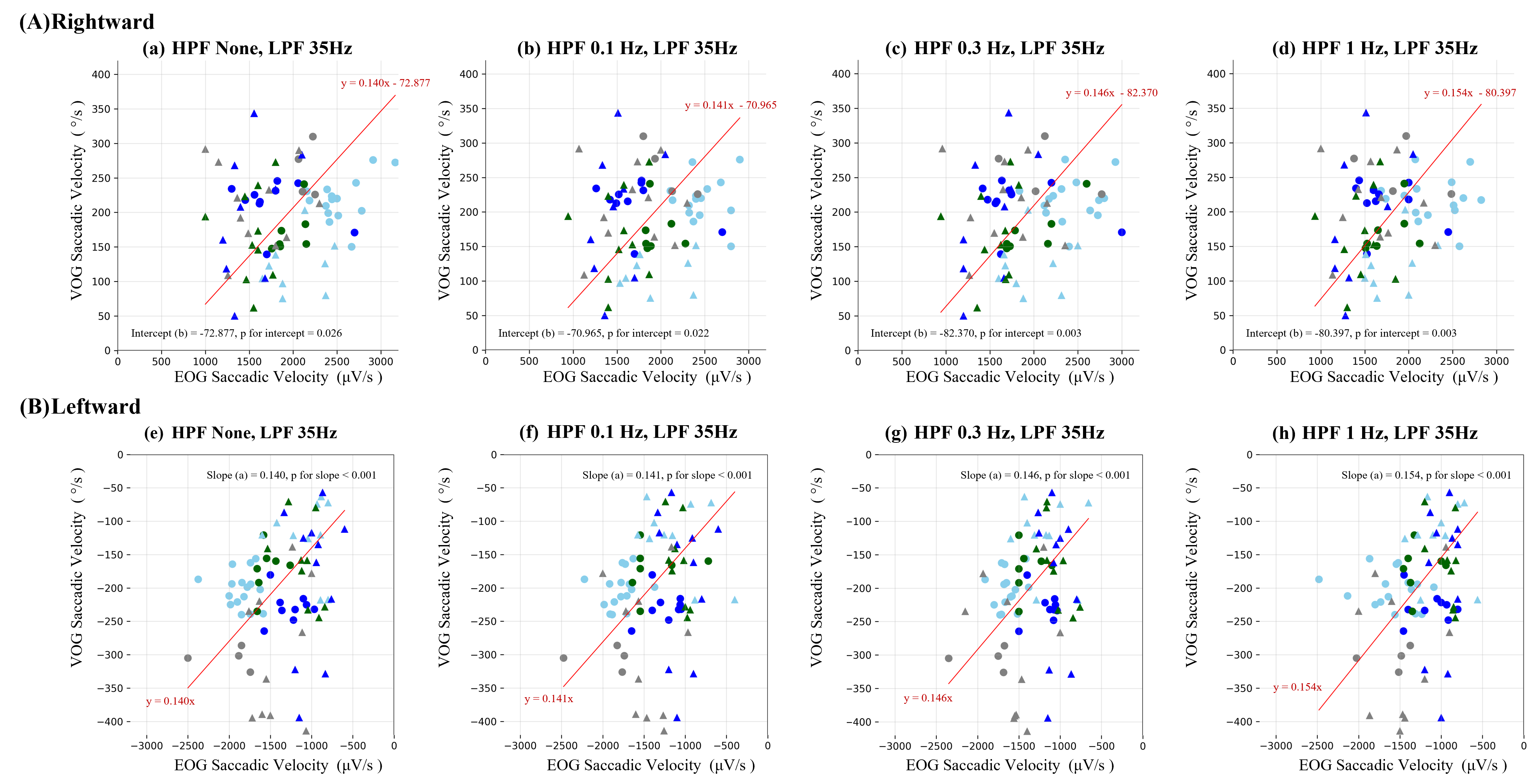
Supplementary Figure S2.** Direction-specific transformation models between EOG- and VOG- derived saccadic velocities under different HPF settings. (A) Rightward; (B) leftward. Data points for Subject 1 (sky blue), subject 2 (blue), subject 3 (green), and subject 4 (gray) are shown. The “●” symbol represents a fixed 40° position, whereas the “▲” symbol indicates random saccades. For leftward saccades, the transformation model was constrained to pass through the origin (y = ax), and the slope (a) was estimated from the leftward data. For rightward saccades, the slope estimated from the corresponding leftward model was fixed, and an intercept term (b) was subsequently estimated (y = ax + b). Red lines represent the fitted transformation models. The reported p-values correspond to tests of estimated slope (a) in the leftward models and the estimated intercept (b) in the rightward models.

**
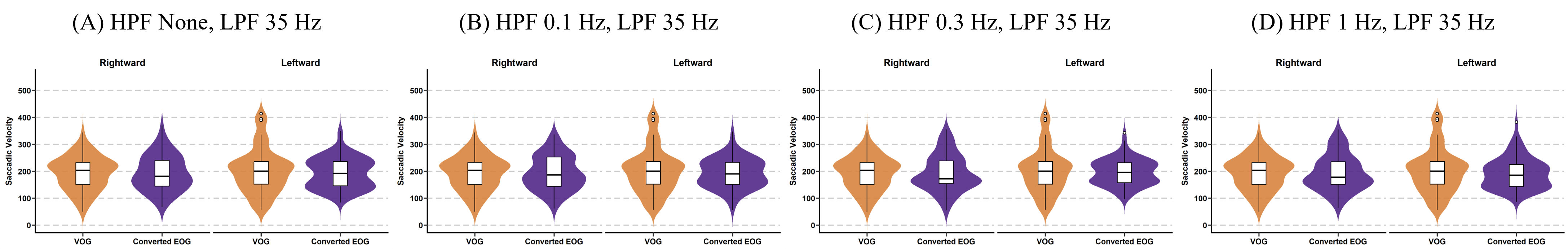
Supplementary Figure S3.** Violin plots comparing saccadic velocities of the right eye between VOG and converted EOG (using the transformation model) under different HPF conditions, with LPF fixed at 35 Hz. Each violin shows the distribution of saccadic velocities; the central white box represents the interquartile range (IQR), and the horizontal line inside the box indicates the median velocity.

## Supplementary Tables

**Supplementary Table S1.**

Comparison of saccadic velocities of the right eye between synthetic and real-world EOG signals across different HPF settings

|  | Synthetic EOG | | Real-world EOG | |
| --- | --- | --- | --- | --- |
|  | Horizontal Saccadic velocity (μV/s) | Relative to HPF None (%) | Horizontal Saccadic velocity (μV/s) | Relative to HPF None (%) |
| HPF None | | | | |
| Rightward | 2061.7 ± 13.8 | 100.0 | 1904.4 ± 473.3 | 100.0 |
| Leftward | $-$1693.2± 7.7 | 100.0 | −1380.9 ± 409.4 | 100.0 |
| HPF 0.1 Hz | | | | |
| Rightward | 2043.9 ± 13.5 | 99.1 | 1882.6 ± 454.2 | 98.9 |
| Leftward | $-$1675.9 ± 8.9 | 99.0 | −1369.0 ± 394.1 | 99.1 |
| HPF 0.3 Hz | | | | |
| Rightward | 1934.2 ± 14.4 | 93.8 | 1891.0 ± 464.7 | 99.3 |
| Leftward | $-$1575.2 ± 8.8 | 93.0 | −1345.5 ± 333.3 | 97.4 |
| HPF 1 Hz | | | | |
| Rightward | 1739.9 ± 13.2 | 84.4 | 1774.8 ± 426.6 | 93.2 |
| Leftward | $-$1390.2 ± 9.6 | 82.1 | −1251.2 ± 376.0 | 90.6 |

Values are presented as mean ± standard deviation (SD). Absolute Saccadic velocity progressively decreased as the high-pass filter (HPF) cut-off frequency increased from none to 1 Hz, showing a generally consistent change in both synthetic and real-world EOG signals.

**Supplementary Table S2.**Comparison of saccadic velocities of the right eye between VOG, EOG and Converted EOG in the derivation dataset under different HPF conditions.

|  | | 4 Subjects (*n* = 151) | | |  |
| --- | --- | --- | --- | --- | --- |
|  |  | VOG-derived velocity  (°/s) | EOG-derived velocity  (μV/s) | Converted EOG-derived velocity  (°/s) | P value |
| Horizontal  Saccadic velocity | | | | | |
| HPF None | Rightward | 193.5 ± 62.3 | 1904.4 ± 473.3 | 193.5 ± 66.2 | 1.000 |
|  | Leftward | $-$203.3 ± 83.3 | −1380.9 ± 409.4 | $-$193.2 ± 57.3 | 0.314 |
| HPF 0.1 Hz | Rightward | 193.5 ± 62.3 | 1882.6 ± 454.2 | 193.5 ± 63.8 | 1.000 |
|  | Leftward | $-$203.3 ± 83.3 | −1369.0 ± 394.1 | $-$192.3 ± 55.4 | 0.292 |
| HPF 0.3 Hz | Rightward | 193.5 ± 62.3 | 1891.0 ± 464.7 | 193.5 ± 67.8 | 1.000 |
|  | Leftward | $-$203.3 ± 83.3 | −1345.5 ± 333.3 | $-$196.3 ± 48.7 | 0.479 |
| HPF 1 Hz | Rightward | 193.5 ± 62.3 | 1774.8 ± 426.6 | 193.5 ± 65.8 | 1.000 |
|  | Leftward | $-$203.3 ± 83.3 | −1251.2 ± 376.0 | $-$193.1 ± 58.0 | 0.310 |

Values are presented as mean ± standard deviation (SD). Statistical comparisons between VOG-derived velocity and converted EOG-derived velocity were conducted using paired t-tests, and the resulting p-values are reported to indicate statistical significance. These results were obtained from a total of 151 velocity measurements from four participants in the derivation dataset.

**Supplementary Table S3.**

Leave-one-subject-out (LOSO) validation of the EOG-to-VOG transformation model.

| Held-out subject | n | Slope (a) | Intercept (b, °/s) | T-test p | Bias  (°/s) | Lower LoA (°/s) | Upper LoA (°/s) | LoA width (°/s) | MAE  (°/s) | RMSE (°/s) |
| --- | --- | --- | --- | --- | --- | --- | --- | --- | --- | --- |
| S1 | 50 | 0.165 | -86.283 | 0.386 | 12.605 | -187.207 | 212.416 | 399.623 | 88.993 | 101.704 |
| S2 | 39 | 0.139 | -82.910 | 0.947 | -1.078 | -196.903 | 194.747 | 391.651 | 81.917 | 98.628 |
| S3 | 33 | 0.149 | -87.548 | 0.621 | -5.762 | -135.851 | 124.326 | 260.177 | 52.405 | 65.612 |
| S4 | 29 | 0.136 | -73.128 | 0.628 | 10.210 | -209.467 | 229.887 | 439.354 | 89.935 | 110.603 |
| **Mean** | **37.75** | **0.147** | **-82.467** | **-** | **3.994** | **-182.357** | **190.344** | **372.701** | **78.313** | **94.137** |
| **All Subjects** | **151** | **0.146** | **-82.370** | **0.609** | **3.529** | **-162.179** | **169.238** | **331.417** | **67.965** | **84.339** |

For each iteration, one participant was excluded from model derivation and used as an independent test subject. The table summarizes the estimated transformation coefficients and validation performance obtained from the held-out participant. Agreement between converted EOG-derived and VOG-derived saccadic velocities was evaluated using Bland-Altman analysis, including the mean bias, lower and upper limits of agreement (LoA), and LoA width. Prediction error was additionally assessed using the mean absolute error (MAE) and root mean square error (RMSE). Statistical comparison between VOG-derived velocities and converted EOG-derived velocities were performed using paired t-test, and the resulting p-values are reported to assess statistical significance. Mean LOSO represents the average performance across the four leave-one-subject-out iterations. All subjects refer to the model derived and evaluated using the complete dataset (n=151).
